# Indents

**Published:** 1903-11-15

**Authors:** 


### INDENTS.

☞ **Brief Articles of Technical or Scientific Value will receive notice and comment. Contributions invited.**

**To prevent Shrinkage in Plaster.** Mix the plaster for pouring with lime water.—X. Dodel, *Pointers.*

**Purifying Wax.** Stir into boiling wax the white of an egg, and it will serve mechanically to clean and purify the wax.—Dr. Van Orden, in *Pacific Gazette.*

**Cleaning Cement Spatulas.** Use very fine emery cloth, such as jewelers use. This will clean the spatula and not scratch it.—*Review.*

**Polishing Gold Fillings.** Don't put too bright polish on gold fillings, as brightly polished fillings may appear to be cavities in artificial light.—*Pacific Gazette.*

**Canal Points.** Pinch the large end of the point with a pair of large flat-nosed pliers. This gives a flat surface to the end which can be firmly grasped with the cotton pliers.—A. W. Thornton, in *Review.*

**Lining for Electric Furnace.** Take equal parts of fused fireclay, silex and fireclay, mix with water, carve to desired form and bake in the furnace.—C. E. Post, in *Pacific Gazette.*

**Sensitive Dentin.** Jarring the tooth with an automatic mallet, having a blunt point plugger in the cavity, aids materially in inducing the penetration of fluids into the dentin.—N. C. Leonard, *Headlight.*

**To Prevent Plaster adhering to Flasks.** Paint the inside of the flask with a thick solution of whiting before pouring the plaster. This prevents corroding and the plaster comes away easily.—*Dental Hints.*

**Insanity from Gas.** A man in Louisville recently went to a dentist who gave him gas to enable him to extract a tooth. Shortly afterward he developed symptoms of insanity which resulted in his transfer to an asylum.

**Separating Medium.** Make a saturated solution of soap, and strain and then add an equal quantity of lard oil and a little coloring matter. Shake well before using and you will have an easy separation.—G. E. Warren, in *Digest.*

**Pressure Anesthesia.** If a pellet of cotton be placed over the rubber in the cavity, a much better pressure can be obtained, as it prevents the rubber from spreading so much under the instrument.—W. A. Brownlee, in *Dominion Journal.*

**The Plaster Bench.** A piece of plate glass about a foot wide and two feet long, set in the plaster bench near the waste drawer, presents a smooth surface on which to set models, and is easily cleaned.—A. W. Thornton, in *Dominion Journal.*

**To Replace a Broken Plaster Tooth on a Model.** Drill a hole in the cast and also in the broken tooth; fill both holes with very thin cement and place in position. This will form a pin of cement securing the tooth in its original position.—J. R. Herzog, *Summary.*

**Sensitive Dentin.** In cavities where the dentin is sensitive I take a pledget of cotton, thrust it into the spirit lamp and let it ignite, and while hot place it in the cavity and leave it there. I find that in many such cases I can cut sensitive dentin very much better after this treatment.—Dr. Austin, *Era.*

**Antidolorin for Diagnosis.** Should there be any doubt if the pulp of a tooth is alive, isolate the tooth in question by covering adjoining teeth with absorbents; spray ethyl chloride on tooth, if pulp is living a reaction will take place at once, if the spray brings no sensation to the tooth the pulp is dead.

**Supports for Baking Porcelain Crowns.** Take pieces of soap-stone and carve them into suitable forms and sized to support crowns and bridges while baking. Then bake them and they will get hard like the lava tips of a gas-burner, and will last a long time.—J. H. Prothero, in *North-Western Journal.*

**Amalgam Mixer.** Take a rubber finger-cot such as is used by surgeons, place the proper proportions of mercury and alloy in the rubber cot, and while closing the opening with one hand knead the mass to the desired consistency. The results are obtained quickly and without soiling the hands.—J. M. Thompson, in *October Cosmos.*

**Inserting Gutta-Percha Points.** Chloroform will cut gutta-percha and permit of its easy penetration of the canals. But a canal so filled, if tested in two or three years, will be noticeably foetied. This would not occur if oil of cajeput were used in place of the chloroform, as it does not disintegrate the gutta-percha.—J. M. Magee, in *Dominion Journal.*

**Antidolorin For extraction of Teeth.** For minor surgery in buccal cavity such as opening an alveolar abscess, removing epules or any operation which can be quickly done, spray parts to be anesthetized until perfectly blanched, and after allowing them to regain their normal color make incision. Always isolate the parts to be anesthetized and get better results with a lesser quantity of the drug.

**Practice not Transferable in Germany.** A physician sold his practice to another and sued for the $475 agreed on, which the buyer had failed to pay. The courts at Braunschweig, where the transaction occured, dismissed the case, claiming that a medical practice is not a marketable commodity, and that a transaction involving its sale is unworthy of the profession and contrary to the best interests of the community.—*Jour. A. M. A.*

**Cleaning Glass Cement Slabs.** Every practitioner knows how difficult it is to remove the cement which adheres to a glass cement slab. Usually a knife is resorted to and the slab presents a scratched appearance therefrom. I accidently discovered that dilute nitric acid will remove all cement particles no matter how hard, and the slab, after being rinsed in water and dried, will have a clean, smooth surface.—George Zederbaum, in *Review.*

**Fundamentals of Artificial Denture.** It should be thoroughly understood that there are at least two fundamentals of artificial dentures, one is the useful or functional and the other is the artistic. These can not always be harmonized in individual cases, then the more important does and should predominate. This should never be taken advantage of to excuse bad workmanship or judgment.—O. A. Weiss, in *Review.*

**Children's Teeth to be Cared For.** According to newspaper reports the municipality of Strasburg, Germany, has voted to build an $80,000 dental hospital for school children. Each pupil must submit to a dental examination on entering and twice during each school year until the age of thirteen. The burghers have come to the conclusion that a large proportion of the ills children suffer from are due to bad teeth and lack of proper dental attention.

**Orthodontia.** In correcting irregularities of the teeth, normal occlusion should be sought in every case. The full complement of teeth is necessary for the best results, and when mutilation has taken place the missing teeth should be substituted with some artificial appliance. The expansion arch and its complementary appliance is better adapted to all regulating work than any other form of appliance.—E. H. Angle, in October *Review*.

**Soda for Sterilizing Root-Canals.** As a sterilizing agent in root-canals, try a fifty percent solution of ordinary caustic soda, applying it just before filling the canal. Great care should be taken not to allow it to come in contact with the lips or gums, as it is a very powerful escharotic. No drying of the canal is necessary. Flush the cavity with alcohol, then with chloroform, and proceed to fill with chloro-percha or gutta-percha points.—G. Chisholm, in *Cosmos*.

**Quick Separation Contra-indicated in Teeth to be Filled with Amalgam.** When amalgam is used, quick wedging is contra-indicated in any but small fillings. Unless the teeth are kept apart sufficiently long to overcome the tendency to immediately resume their normal position, any contouring will be destroyed possibly, indeed, the filling may be broken up and disintegrated by the teeth springing together as soon as the wedge is removed.—D. Lindley Palmer, *Dental Brief*.

**Handy Soldering Block.** Take about a yard of asbestos rope which may be had of any hardware store, and coil it after the manner of a rope on a boat deck.

This will make a flat coil of three or four inches in diameter. Cover this to the thickness of one inch with a mixture of plaster of paris and pumice, with a few shreds of asbestos fiber. While the plaster is still soft, insert three nails for legs to rest it on. The nails should be about three inches long and set in the form of a triangle.—J. W. Hogey, in *Dominion Journal*.

**Removing Pulps.** Recently I had occasion to remove a pulp from the left first upper bicuspid and upper right first molar. I placed a small pledget of cotton, saturated with a 50 percent cocain solution, in the molar cavity directly upon the exposed pulp. To prevent leaking into the mouth I covered it with a soft filling of cotton, making a slight pressure. I then placed the rubber dam on another tooth and removed its pulp by the pressure method, and then came back to the molar and found it insensible so that I removed the pulp without pain.

**Retaining Splint.** A splint for retaining loose teeth may be made by banding with 22-karat gold plate. After the several bands are soldered together, an impression is taken with the bands upon the teeth, with modeling composition, into which ideal fusible alloy is cast to make a die. Modeling composition is used for the counter-die to swage a 22-karrat 30-gauge gold back, which is soldered to the lingual surface of the bands; the distal part of the bands is then cut away, leaving a wedge-shaped piece between the teeth.—Dr. W. H. Todd, in *Review*.

**Sterilizing Instruments.** Place a ring bracket on the wall near the cabinet and place in it a large glass tumbler, fill this half or two-thirds full of soft water and add to it a teaspoonful of formalin. After using instruments wipe off accumulations and stand them in this solution. They can be removed at any time after fifteen minutes and wiped dry and used again or put away in the case. —H. A. Jelly, in *Cosmos*. A more useful sterilizing medium would be a two percent solution of lysol. The instruments can remain in it for hours without rusting or corroding.—*Ed. Register*.

**Poisoning with the Powerful Antiseptics.** Remember that mercury bichlorid, carbolic acid, and iodoform may cause systemic poisoning, even in the small amounts commonly employed for dressings. The sublimate gives rise to diarrhea with pain and a rising temperature; carbolic acid poisoning is attended by the well-known changes in the urine, sometimes with severe vomiting, lowered temperature and collapse; while iodoform may cause collapse or a rise of temperature, with a feeble pulse, and delirium and drowsiness, particularly in children.—*International Journal of Surgery*.

**Investment Material.** An investment material for bridge-work, that will not check or break, even under rough treatment, may be made by taking long fiber asbestos (purchased from a dealer in plumbers' supplies in the form of an asbestos rope), picking it to pieces with the fingers, and placing the fiber in the water, to which the plaster of paris is afterward added. This investment dries out very quickly, will not change form and never requires to be wired or banded. It is inexpensive and one or two trials will enable any one to determine the proper proportion of water, asbestos fiber and plaster.—Arthur G. Smith, in *Review*.

